# Phononic and magnonic dispersions of surface waves on a permalloy/BARC nanostructured array

**DOI:** 10.1186/1556-276X-8-115

**Published:** 2013-03-02

**Authors:** Huihui Pan, Vanessa Li Zhang, Kai Di, Meng Hau Kuok, Hock Siah Lim, Ser Choon Ng, Navab Singh, Adekunle Olusola Adeyeye

**Affiliations:** 1 Department of Physics, National University of Singapore, Singapore 117542, Singapore; 2 Institute of Microelectronics, Agency for Science, Technology and Research, Singapore 117685, Singapore; 3 Department of Electrical and Computer Engineering, National University of Singapore, Singapore 117576, Singapore

**Keywords:** Phononic and magnonic dispersions, Bandgaps, Brillouin light scattering, Magphonic crystal

## Abstract

Phononic and magnonic dispersions of a linear array of periodic alternating Ni_80_Fe_20_ and bottom anti-reflective coating nanostripes on a Si substrate have been measured using Brillouin light scattering. The observed phononic gaps are considerably larger than those of laterally patterned multi-component crystals previously reported, mainly a consequence of the high elastic and density contrasts between the stripe materials. Additionally, the phonon hybridization bandgap has an unusual origin in the hybridization and avoided crossing of the zone-folded Rayleigh and pseudo-Sezawa waves. The magnonic band structure features near-dispersionless branches, with unusual vortex-like dynamic magnetization profiles, some of which lie below the highly-dispersive fundamental mode branch. Finite element calculations of the phononic and magnonic dispersions of the magphonic crystal accord well with experimental data.

## Background

Photonic-phononic crystals, also referred to as phoxonic crystals [1-4], are of great interest as their dual photonic and phononic bandgaps allow the simultaneous control of photon and phonon propagation in these crystals. Another class of metamaterials possessing dual-excitation bandgaps is magnonic-phononic or magphonic crystals [5-7]. Although less well known than phoxonic materials, they too have promising application potential because of the possibility of the simultaneous control and manipulation of magnon and phonon propagation in them. Hence, they are potentially more useful technologically than either solely magnonic or phononic crystals which depend on a single type of excitation, namely magnons or phonons, as the respective information carrier.

Magphonic crystals were theoretically studied by Nikitov et al. in 2008 [5]. Recently, Zhang et al. experimentally studied these materials in the form of a two-dimensional (2D) chessboard-patterned array of cobalt and Ni_80_Fe_20_ (Permalloy, Py) dots [6], and one-dimensional (1D) periodic arrays of alternating Fe (or Ni) and Py nanostripes on SiO_2_/Si substrates (henceforth referred to as Py/Fe(Ni)) [7]. As the materials of the elements of these bicomponent arrays are both metals, namely either Py/Co, Py/Fe, or Py/Ni, the elastic and density contrasts between adjacent elements are rather low.

In general, the phononic bandgap width increases with elastic and density contrasts [8,9]. Indeed the phonon bandgaps of the 1D and 2D structures measured by Zhang et al. are small, being of the order of 0.5 GHz. In this work, the magphonic crystal studied is a 1D periodic array of alternating Py and bottom anti-reflective coating (BARC) nanostripes deposited on an Si(001) substrate (abbreviated to Py/BARC). Py and BARC were selected as materials for the high elastic and density contrasts between them. Hence, the phononic dispersion is expected to be significantly different from those of Py/Fe(Ni). It is also of interest to explore the effects on the magnonic dispersion when the material of one of the elements in a bicomponent magphonic crystal is a non-magnetic one.

The dispersions of surface spin and acoustic waves were measured by Brillouin light scattering (BLS) which is a powerful probe of such excitations in nanostructured materials [6,7,9-13]. The measured phononic dispersion spectrum features a Bragg gap opening at the Brillouin zone (BZ) boundary, and a large hybridization bandgap, whose origin is different from those reported for other 1D-periodic phononic crystals [6,13-16]. Interestingly, the experimental magnonic band structure reveals spin wave modes with near-nondispersive behavior and having frequencies below that of the highly dispersive fundamental mode (see below). This differs from the 1D one- or two-component magnonic crystals studied earlier, where almost dispersionless branches appear well above the dispersive branches [6,12]. Numerical simulations, carried out within the finite element framework, of the phononic and the magnonic dispersions yielded good agreement with experiments.

## Methods

### Sample fabrication

**Figure 1:**
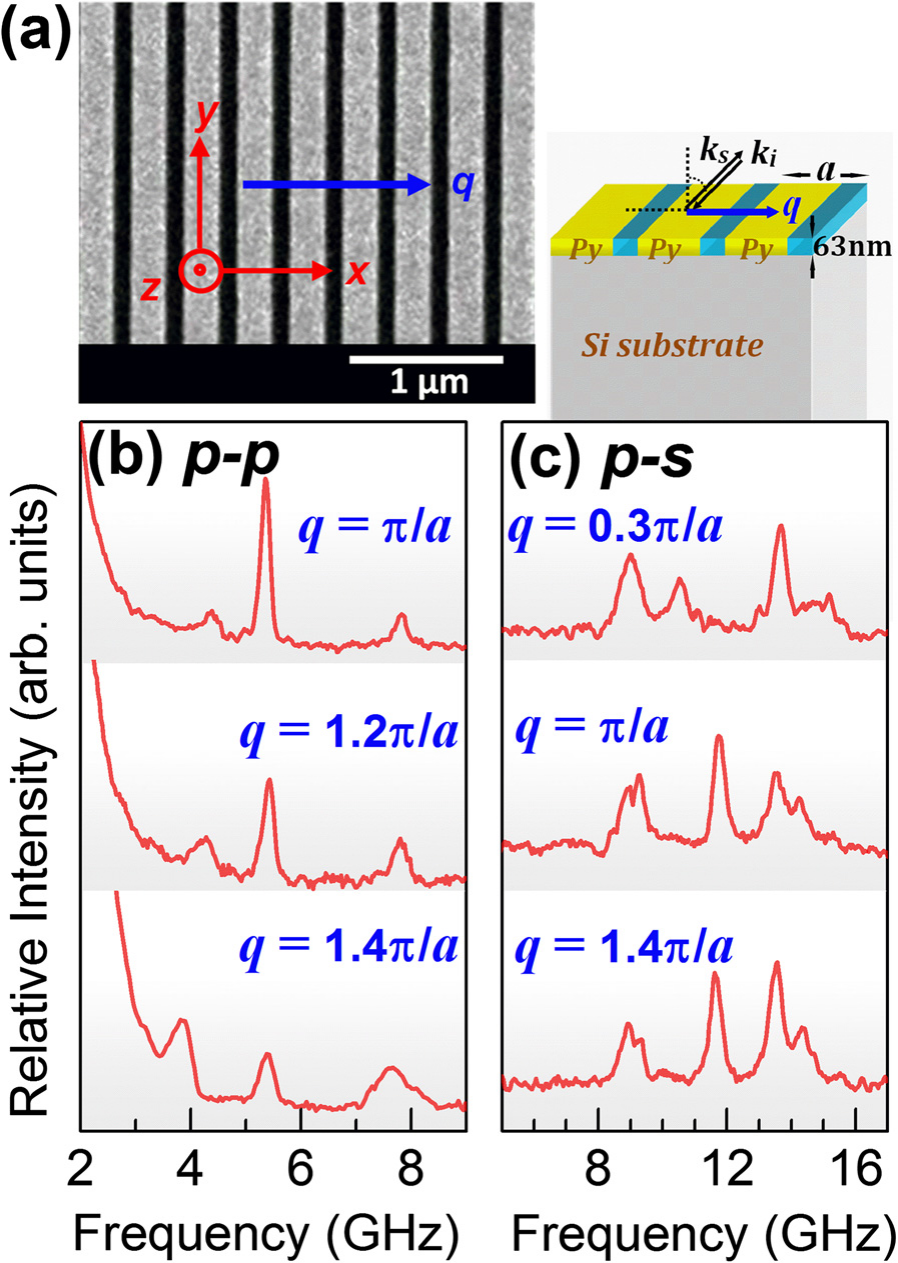
**SEM image and Brillouin spectra of the Py/BARC magphonic crystal.** (**a**) SEM image and schematics of the sample and scattering geometry employed, showing the orientation of the Cartesian coordinate system with respect to nanostripes and phonon/magnon wavevector ***q***. Polarization Brillouin spectra of (**b**) phonons and (**c**) magnons. Lattice constant *a* = 350 nm.

A 4 × 4-mm^2^-patterned area of 63 nm-thick 1D periodic array of alternating 250 nm-wide Py and 100 nm-wide BARC nanostripes (lattice constant *a* = 350 nm) was fabricated on a Si(001) substrate using deep ultraviolet (DUV) lithography at 248 nm exposing wavelength [17]. The substrate was first coated with a 63-nm-thick BARC layer, followed by a 480-nm-thick positive DUV photoresist. A Nikon lithographic scanner with a KrF excimer laser radiation was then used for exposing the resist. To convert the resist patterns into nanostripes, a 63-nm-thick Py was deposited using electron beam evaporation technique followed by the lift-off in OK73 and isopropyl alcohol. An ultrasonic bath was used to create agitation for easy lift-off of the Py layer. Completion of the lift-off process was determined by the color contrast of the patterned Py regions and confirmed by inspection under a scanning electron microscope (SEM). Figure 1a shows an SEM image of the resulting structure.

### Brillouin measurements

**Figure 2:**
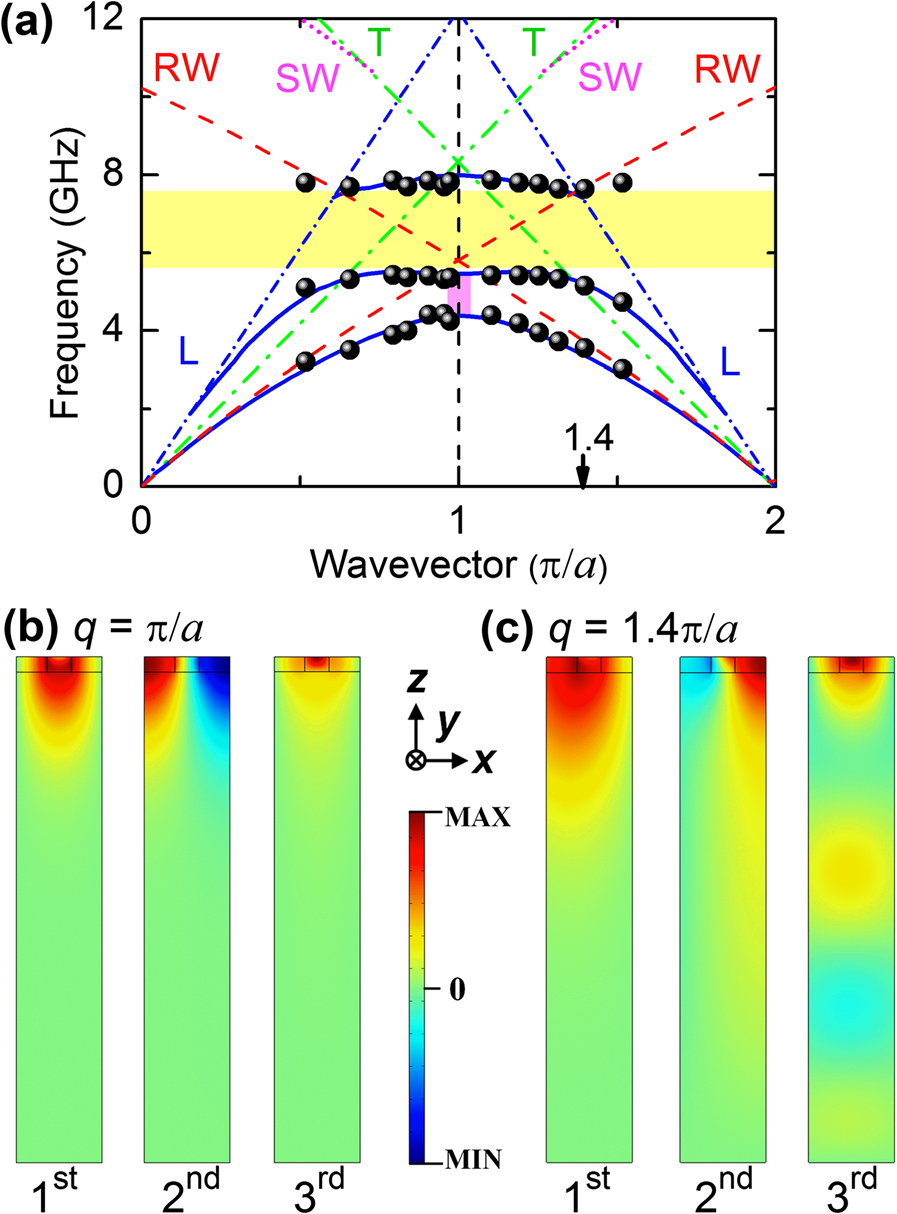
**Phonon dispersion relations and mode displacement profiles.** (**a**) Phonon dispersion relations of the Py/BARC magphonic crystal. Experimental and theoretical data are denoted by dots and solid lines, respectively. Red-dashed lines and magenta-dotted lines represent the simulated Rayleigh wave (RW) and Sezawa wave (SW) dispersions for the effective medium film on Si(001) substrate. The transverse (T) and longitudinal (L) bulk wave thresholds are represented by respective green dot-dashed lines and blue short-dot-dashed lines. Measured Bragg gap opening and the hybridization bandgap are indicated by a pink rectangle and a yellow band, respectively. *z*-components of the displacements of observed phonon modes at (**b**) *q* = π/*a* and (**c**) *q* = 1.4π/*a*.

**Figure 3:**
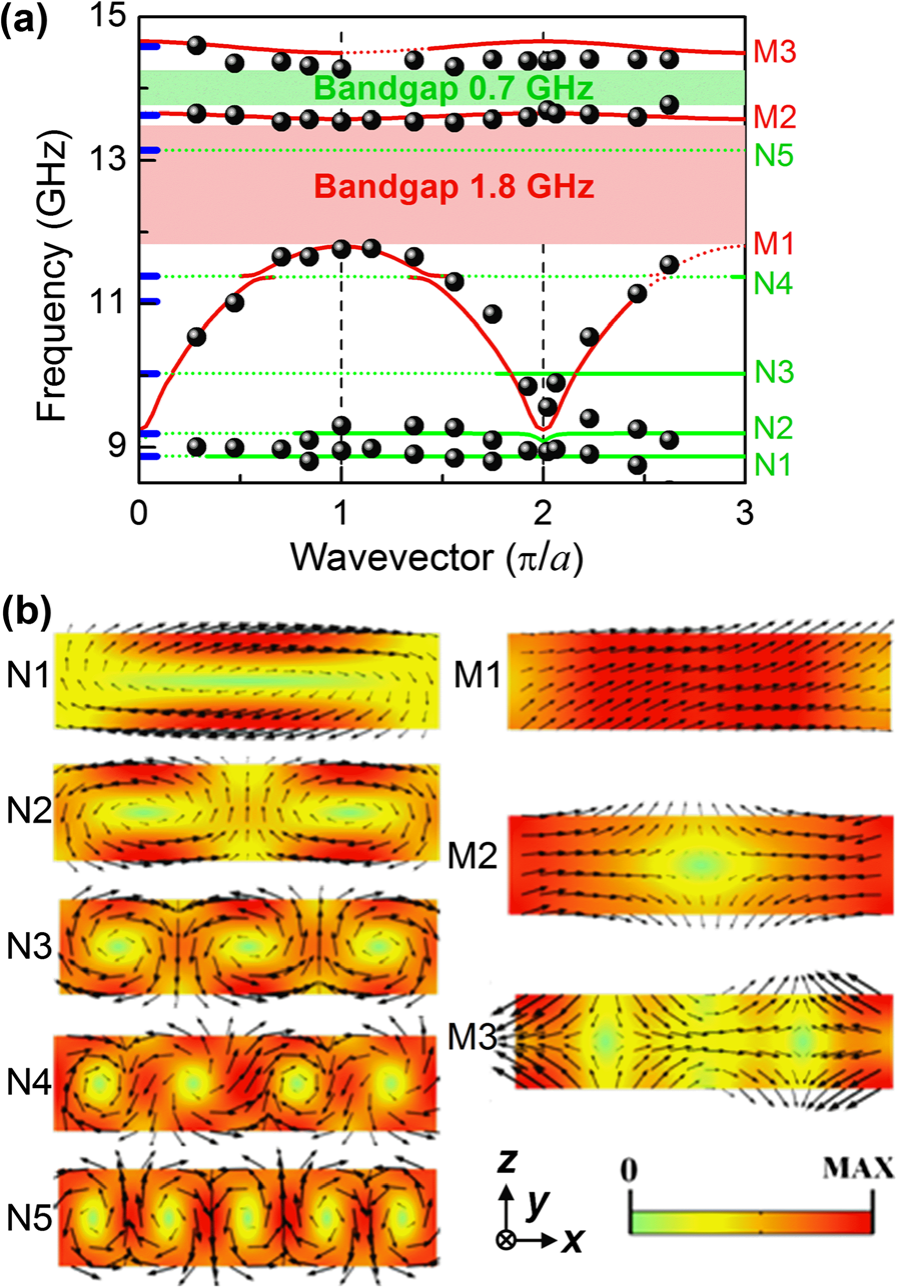
**Magnon dispersion relations and magnetization profiles.** (**a**) Magnon dispersion relations of the Py/BARC magphonic crystal. Experimental data are denoted by dots and theoretical data by lines, with solid (dotted) lines representing modes with relatively strong (weak) intensities. Measured bandgaps are shown as shaded bands, and Brillouin zone boundaries as vertical-dashed lines. The theoretical branches are labeled M1 to M3 and N1 to N5 (see text). The blue bars around *q* = 0 indicate calculated frequencies of the confined modes of an isolated Py stripe. (**b**) Cross section of magnetization profiles of the magnon modes within one Py stripe in a unit cell of the magphonic crystal at *q* = π/*a*. The dynamic magnetization vectors are represented by arrows, with their color-coded magnitudes.

The 180°-backscattering geometry was used in the BLS experiments, with the scattering plane normal to the sample surface and the magnon or phonon wavevector ***q*** along the periodicity direction (*x* direction in Figure 1a) which coincides with the [110] direction of the Si substrate. The 514.5-nm radiation of an argon-ion laser served as the light source and the scattered light was frequency analyzed with a (3 + 3)-pass tandem Fabry-Pérot interferometer equipped with a silicon avalanche diode detector. Prior to the spectral scans, the sample was first saturated in a 0.7-tesla field applied along the symmetry axes of the stripes, which was then gradually reduced to zero. Spectra of the acoustic and spin waves were measured in the *p-p* and *p-s* polarizations, respectively, and their dispersion relations mapped by varying the laser light incidence angle. Figure 1b,c shows typical Brillouin spectra recorded for the two excitations. Their mode frequencies obtained from spectral fits using Lorentzian functions were plotted against wavevector to yield dispersion relations shown in Figures 2a and 3a.

## Results and discussion

We will first focus our attention on the phononic dispersion. The measured phononic dispersion spectrum features a 1.0-GHz gap opening centered at 4.8 GHz at the Brillouin zone boundary, and a 2.2-GHz bandgap centered at 6.5 GHz. Dispersion relations and mode displacement profiles of surface acoustic waves (SAWs) were computed using the finite element approach in COMSOL Multiphysics [18] and the Bloch-Floquet theorem. The 350-nm-wide computational cell used comprises a 63-nm-thick layer of a 100-nm-wide BARC stripe sandwiched between two 125-nm-wide Py stripes, atop a 2-μm-thick Si substrate, with its bottom boundary fixed. It is to be noted that unlike the case of the 1D Py/Fe nanostripe array of [7], no interfacial air gaps were considered in the calculations, as the fabrication process employed here precludes their formation. Elastic parameters used in the simulations for Py, BARC, and Si are Young’s moduli = 180, 6.26, and 169 GPa; Poisson ratios = 0.31, 0.34, and 0.064; and mass densities = 8600, 1190, and 2330 kg/m^3^, respectively [19-21]. The simulated dispersion relations for the lowest three SAW branches, below the longitudinal bulk wave threshold [22,23], presented in Figure 2a, accord well with the Brillouin measurements. Also shown in the figure are the dispersion relations of the vertically polarized transverse (T) and longitudinal (L) bulk waves, in the [110] direction, of the Si substrate.

Simulated mode profiles for *q =* π*/a*, shown in Figure 2b, of the lowest two modes exhibit characteristics of the surface Rayleigh wave (RW). These RWs are standing Bloch waves satisfying the Bragg scattering condition. The mode profile of the third branch at the BZ boundary reveals that it is also a standing wave with most of its energy confined in the BARC stripes. Mode profiles for *q =* 1.4π*/a* displayed in Figure 2c indicate that at this wavevector, the first branch has the characteristics of the RW. In contrast, the higher two SAWs leak energy into the Si substrate as their dispersion curves extend beyond the transverse bulk wave threshold [16,22-24]. The dispersion relations of the RW and Sezawa wave (SW), modeled by treating the Py/BARC array as a homogeneous effective medium [25] on a Si substrate, are presented in Figure 2a. It can be seen that the gap opening arises from the zone folding of the RW dispersions and avoided crossings at the BZ boundary.

A prominent feature of the phonon dispersion spectrum is the large hybridization bandgap. For a structure, such as ours, comprising a ‘slow’ film on a ‘fast’ substrate, Sezawa waves will exist only below the transverse bulk wave threshold, and over a restricted range of *qh*, where *h* is the film thickness [23,26]. As shown in Figure 2a, within the first BZ, the SW and zone-folded RW do not cross, indicating that the measured bandgap does not originate from the hybridization of these waves. Instead, within the bandgap, the zone-folded RW crosses the transverse bulk wave threshold. Additionally, above but close to this threshold, attenuated SAWs called pseudo-Sezawa waves which exist as resonances with the substrate continuum of modes have been observed [23,26,27]. We thus attribute the origin of the bandgap to the hybridization and avoided crossing of the zone-folded RW and pseudo-Sezawa waves. The origin of this hybridization bandgap is to be contrasted with those reported for other 1D phononic crystals. For instance, Zhang et al. [7] and Maznev [15,16] attributed the origin of the gaps they observed in film-substrate samples to the avoided crossings of the RW and zone-folded Sezawa modes. Also, hybridization bandgaps in Si and SiO_2_ gratings [13,14] were ascribed to the mixing of the RW and the longitudinal resonance, also referred to as the high-frequency pseudo-surface wave.

It is noteworthy that the phonon dispersion spectrum of Py/BARC differs substantially from those of the 1D Py/Fe(Ni) arrays of [7]. For instance, the measured gap opening of 1.0 GHz at the BZ boundary of the former, is much wider than the first bandgap of 0.4 GHz observed for the latter. This is primarily due to the elastic and density contrasts between two metals (Fe or Ni and Py) being much lower than that between the polymer BARC and the metal Py. The 4.8 GHz center of this gap opening is also higher than those (≈ 3.4 GHz) of Py/Fe(Ni). This is expected as the 350-nm period of our Py/BARC is shorter than the 500-nm one of Py/Fe(Ni). Another reason is that our Py/BARC is directly patterned on a Si substrate, while the Py/Fe(Ni) samples contain an 800-nm-thick SiO_2_ sub-layer between the patterned arrays and the Si substrate which has the effect of red shifting the SAW frequencies. Another notable difference is that the 2.2-GHz bandgap is considerably larger than those of the Py/Fe(Ni) arrays, whose maximum gap is only 0.6 GHz. One explanation for this is the high elastic and density contrasts between the materials in Py/BARC.

We now discuss the dispersion of spin waves in Py/BARC. The magnon band structure (Figure 3a) and mode profiles of the dynamic magnetization (Figure 3b) were calculated by solving the coupled linearized Landau-Lifshitz equation and Maxwell's equations in the magnetostatic approximation using a finite element approach [10]. As Py has negligible magnetic anisotropy, the free-spin boundary condition [28] is imposed on the Py surface. The Bloch-Floquet boundary condition is applied along the periodic direction. Parameters used for Py are the saturation magnetization *M*_S_ = 7.3 × 10^5^ A/m, the exchange stiffness *A* = 1.2 × 10^-11^ J/m, and the gyromagnetic ratio *γ* = 190 GHz/T. The relative BLS intensities *I* of the magnon modes [11] were estimated from *I* ∝ | ∫ _0_^*a*^*m*_*z*_(*x*)exp(−*iqx*) *dx*|^2^. The dispersion curves of the more intense modes are indicated by bold solid lines while those of weaker ones by dotted lines in Figure 3a, which reveals generally good agreement between experiment and simulations. Aside from the fundamental mode branch, labeled M1 in Figure 3a (see below), the other branches are rather flat.

The magnon eigenmodes of a single isolated Py stripe having the same dimensions as those of a Py stripe in Py/BARC were also calculated using the above approach. Their calculated frequencies are indicated by blue bars in Figure 3a. It can be seen that, except for the fundamental mode branch, the magnon dispersion relation of Py/BARC is similar to that of the isolated Py stripe. In contrast to the magnon band structures of arrays of Py stripes separated by air gaps studied earlier [12], near-dispersionless modes exist below the fundamental mode branch (M1) of our Py/BARC sample. One reason is that the Py stripes in our sample are thicker. In comparison to the Py/Fe(Ni) structures [7], Py/BARC has a generally less-dispersive magnon band structure; however, its measured 1.8 GHz first and 0.7 GHz second bandgaps are of the same order of magnitude as those of the former.

It is to be noted that the magnon branches can be classified into two groups. One group comprises branches (labeled M1 to M3 in Figure 3a) whose modes have profiles that are similar, i.e., near-uniform across the Py stripe thickness (*z* direction), to those observed in Py/air stripe arrays [12,29]. The other dispersionless group (labeled N1 to N5) comprises the perpendicular standing spin waves (PSSW). The frequencies of these PSSW modes, with quantization numbers *n* = 1 and *m* = 0 to 4 across the thickness and width, respectively, were also analytically calculated [11] and found to be 8.64, 8.94, 9.78, 11.1, and 12.8 GHz, in good agreement with experiment. It is noteworthy that the dynamic magnetizations (represented by arrows in Figure 3b) of the PSSW modes form one or more closed loops, each resembling the vortex configuration of a ferromagnetic ring [30]. As the dipolar field outside a magnetic vortex vanishes, the dipole-dipole coupling between the PSSW modes is expected to be very weak. This is evidenced by their nearly flat dispersion curves.

Interestingly, mode hybridizations exist between the fundamental mode M1 and the respective PSSW modes N2 and N4, as borne out by the simulated hybridized mode profiles. Hybridization of the fundamental mode M1 with the N3 mode is however precluded due to their different symmetries. The M1 mode possesses odd symmetry, as under a π-rotation about the symmetry axis (*y* direction) of a Py stripe, its dynamic magnetizations are reversed. The N2 and N4 modes have odd symmetry, while the N3 mode has even symmetry.

## Conclusions

In summary, we have measured the simultaneous magnonic and phononic bandgaps of the Py/BARC magphonic crystal by Brillouin light scattering. The measured phononic Bragg gap opening and hybridization bandgap are much wider than those previously observed in laterally patterned multi-component phononic crystals. This is mainly ascribed to the high elastic and density contrasts between the stripe materials, Py and BARC. The hybridization bandgap is found to have an unusual origin in the hybridization and avoided crossing of the zone-folded Rayleigh and pseudo-Sezawa waves. The magnonic dispersion relation comprises near-dispersionless PSSW branches, with some of them lying below the highly dispersive fundamental mode branch. Modes of the former have interesting vortex-like dynamic magnetization profiles, suggesting that interactions between the Py stripes are weak, and hence accounting for the nearly flat dispersion curves of these modes. Finite element simulations generally reproduced the experimental phonon and magnon dispersion relations. Because of the possibility of simultaneously controlling and manipulating the magnon and phonon propagation in them, magphonic crystals could find applications in areas such as acoustic and spin-wave signal processing.

## Competing interests

The authors declare that they have no competing interests.

## Authors' contributions

HHP performed the experiments, calculations, and analyses of the phononic part as well as drafted the manuscript of this part. VLZ carried out the experiments with HHP and participated in the analyses of both the phononic and magnonic parts. KD carried out the calculations, analyses, and manuscript drafting of the magnonic part. HSL participated in the analyses. MHK and SCN conceived the project and assisted in the interpretation of the results and drafting of the manuscript. AOA and NS fabricated the sample. All authors read and approved the manuscript.
